# Massive gallstone in an asymptomatic Indigenous Canadian male: Case report and literature review

**DOI:** 10.1016/j.ijscr.2020.06.028

**Published:** 2020-06-11

**Authors:** Angela W. Chan, Rathi M. Sabaratnam, Yagan Pillay

**Affiliations:** a 103 Hospital Drive, Royal University Hospital, Department of Surgery, University of Saskatchewan, Saskatoon, SK, S7N 0W8, Canada; b Department of Pathology and Laboratory Medicine, Victoria Hospital- 1200 24th St W, Prince Albert, SK, S6V 4N9, Canada; c University of Saskatchewan, Victoria Hospital- 1200 24th St W, Prince Albert, SK, S6V 4N9, Canada

**Keywords:** Case report, Cholelithiasis, Prophylactic cholecystectomy, Biliary disease, Gallbladder carcinoma

## Abstract

**Introduction:**

Gallstones are present in 10–15% of North Americans, but only 20% become symptomatic. This case report describes a patient with a 7.5 cm gallstone detected incidentally while being investigated for cardiac pathology. This is the first report in the English literature of a stone this size that has remained clinically asymptomatic. This work is reported in line with the SCARE criteria.

**Case report:**

The patient was a 71-year-old Indigenous Canadian male, with atrial fibrillation. His cardiologist ordered a CT scan of his chest, which incidentally identified a large gallstone. He was referred to surgery for asymptomatic cholelithiasis. An uncomplicated laparoscopic cholecystectomy was performed. Final pathology showed a 7.5 cm gallstone with features of chronic cholecystitis.

**Discussion:**

The patient’s ethnicity and gallstone size placed him at increased risk for gallbladder cancer, gallstone fistulization and perforation. We reviewed the literature for asymptomatic patients who may benefit from cholecystectomy: transplant recipients and those with hemolytic disorders. Laparoscopic cholecystectomy is not currently indicated in diabetics and bariatric surgery patients.

**Conclusion:**

This case report shows that there are asymptomatic patients with massive gallstones. A review of their history, risk factors for malignancy and future gallstone related complications must be carefully weighed and discussed prior to deciding on surgical versus expectant management.

## Highlights

• Gallstone size correlates with gallbladder malignancy, fistulization, perforation.
• A rare case of an asymptomatic patient with a 7.5 cm gallstone is presented.
• Surgery is usually offered to surgical candidates with symptomatic cholelithiasis.
• Cholecystectomy can be offered to transplant and hemolytic disorder patients.
• No indications for cholecystectomy in diabetics and bariatric surgery patients.

## 1 Introduction

Cholelithiasis occurs in 10–15% of North American adults. Twenty percent of these individuals become symptomatic [1]. Risk factors for stone formation include age greater than forty years, female sex, obesity, total parenteral nutrition, and pregnancy. Uncomplicated gallstone disease refers to biliary colic. Complicated gallstone disease infers cholecystitis, pancreatitis, choledocholithiasis, ascending cholangitis, cholecystoenteric fistulae, or perforation. The typical symptoms of abdominal pain, nausea, vomiting, jaundice, and even bowel obstruction are related to the specific types of gallstone complication. The current standard of care is to offer a cholecystectomy to surgical candidates who are symptomatic from their gallstones, either emergently or electively.

Larger stones have a greater potential to obstruct bile outflow, leading to biliary colic, acute cholecystitis or obstructive jaundice. The pressure from larger gallstones can perforate the gallbladder or even fistulize into adjacent viscera [2]. There is also a positive correlation between stone size and gallbladder carcinoma [3]. Despite this association between size and symptoms, not all patients with gallstones larger than 2 cm are symptomatic. We present the case of a patient with a 7.5 cm gallstone, discovered incidentally. This is the first reported case in the English literature of a stone this size that has remained asymptomatic. There are no current guidelines for cholecystectomy in asymptomatic patients however in patients with certain risk factors, surgery can be considered. The clinical decision-making process will be discussed in this article.

This work has been reported in line with the SCARE criteria [4].

## 2 Presentation of case

**Fig. 1:**
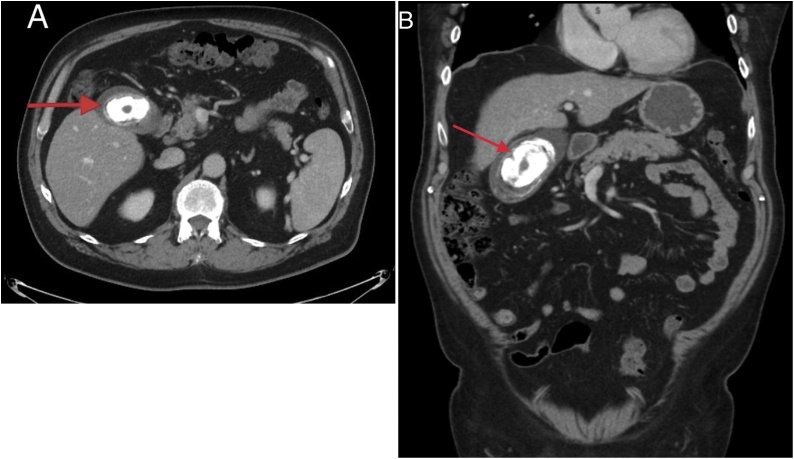
Axial (a) and coronal (b) abdominal/pelvic CT images showing large calcified gallstone in gallbladder.

A 71-year-old Indigenous Canadian male was referred to surgery by his family physician for a massive gallstone. The patient denied any abdominal pain, nausea, vomiting, or jaundice. He had atrial fibrillation and underwent previous electrical cardioversions and a cardiac ablation procedure. Despite best medical treatment, his arrhythmia persisted. His cardiologist ordered a computed tomography (CT) scan of the chest to rule out structural causes of his fibrillation. In addition to interstitial lung disease, a 7.5 cm gallstone was incidentally seen on his chest CT. The remainder of the abdomen and pelvis were subsequently imaged (Fig. 1).

Despite its size, the patient denied any abdominal symptoms and underwent an ultrasound as a follow-up test to his CT. This showed gallbladder wall thickening of approximately 8 mm without any pericholecystic fluid. The heavy shadowing from the large stone made it difficult to exclude a soft tissue mass. The intrahepatic and extrahepatic bile ducts were of normal caliber. A complete blood count and liver enzyme panel were normal.

Past medical history included cataract lens surgery, hypertension and benign prostatic hyperplasia. The patient is a non-smoker and his medications include: Apixaban anticoagulation (Factor Xa inhibitor), Sotalol (beta blocker), Tamsulosin (alpha-1 receptor antagonist), and Perindopril (angiotensin converting enzyme inhibitor). Family history was medically unremarkable. The patient never had any symptoms of abdominal pain or jaundice. Physical examination of his abdomen was soft and non-tender with no palpable masses. There was no jaundice.

The surgeon had an extensive discussion with the patient and his family about possible complications of gallstone disease including cholecystitis, gallstone fistulization and perforation. The risk of malignancy was also discussed, given the size of the stone and his Indigenous heritage. The options of watchful waiting versus elective surgery were presented and he decided upon surgery. Informed consent was obtained for a laparoscopic, possible open cholecystectomy with the use of de-identified patient data for research purposes. He was seen in the pre-admission clinic by an internist and an anesthetist for optimization of his comorbidities. Apixaban was held 48 h prior to surgery and bridging anti-coagulation was not required.

An uncomplicated elective laparoscopic cholecystectomy was performed at Victoria Hospital in Prince Albert, Canada by the surgeon and surgical fellow. Victoria Hospital is a community practice setting. The patient was discharged home on the same day. He was instructed to resume his Apixaban on post-operative day one.

**Fig. 2:**
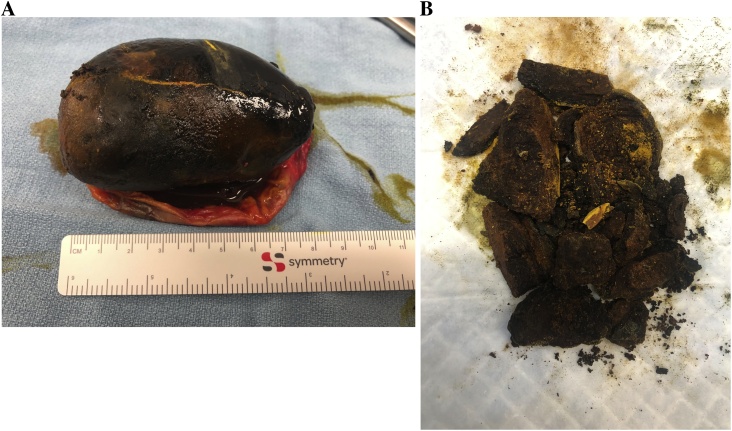
Ex-vivo photograph of 7.5 cm gallstone outside of gallbladder (a) and cut gallstone, with fragments (b).

**Fig. 3:**
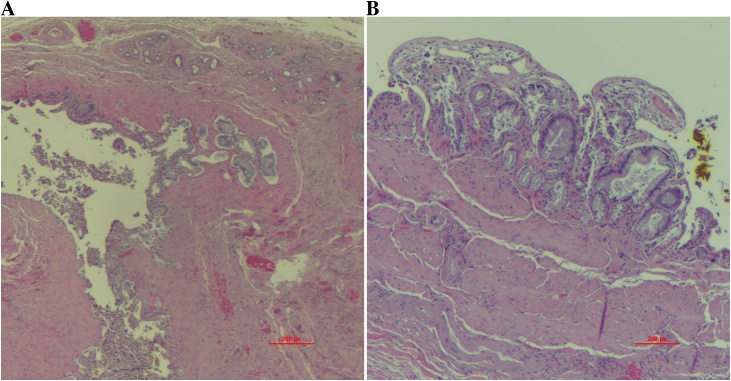
Chronic cholecystitis with mucosal dips, forming Rokitansky-Aschoff sinus with muscular hypertrophy and fibrosis, and minimal chronic inflammation (a). Mild mucosal hyperplasia with surface epithelial denudation and muscle hypertrophy and fibrosis (b).

The calculus measured 7.5 cm in its greatest dimension (Fig. 2a). The stone was cut longitudinally (Fig. 2b) but as it was brittle, it fractured into numerous pieces. Final pathology revealed a gallbladder wall measuring up to 0.1 cm in thickness with features of chronic cholecystitis (Fig. 3a/b). Post-operatively the patient had an uneventful course and was seen in follow-up two weeks after cholecystectomy. He was pain-free, tolerating a regular diet, and all surgical incisions were healed.

## 3 Discussion

We present the first case of an asymptomatic gallstone of this size (7.5 cm). A Pubmed literature search using the keywords “giant gallstone”, “large gallstone” revealed that there were very few cases of gallstones larger than the one found in our patient; of these cases, all patients were symptomatic. A Chilean man with a 16.8 cm stone was having two days of right upper quadrant abdominal pain before he underwent emergency cholecystectomy [5]. A female patient in the United Kingdom had a 10 cm stone, but she presented as a large bowel obstruction secondary to a cholecystocolonic fistula.

For patients with symptomatic cholelithiasis, laparoscopic cholecystectomy remains the standard of care. It is offered to surgical candidates either emergently or electively depending on whether they have biliary colic or complications of gallstone disease. In patients with asymptomatic gallstones, prophylactic cholecystectomy is not usually indicated, but in patients at increased risk of gallbladder cancer or hemolytic disorders an argument can be made for a surgical intervention.

Gallstone presence and size are the main risk factors for malignancy, as subsequent malignant transformation is thought to be due to chronic inflammation from stones. The incidence of gallbladder cancer is seven times more common in people with gallstones [3]. The risk of carcinoma is ten fold in patients with gallstones greater than 3 cm compared to patients with no stones. Some surgeons operate prophylactically for stones greater than 2.5 cm [6,7]. Additional risk factors for gallbladder cancer such as sex, ethnicity, the presence of an anomalous pancreaticobiliary junction, presence and size of gallbladder polyps should be added to the decision-making algorithm when considering surgical intervention.

In Canada, First Nations or Indigenous individuals have a higher incidence of gallbladder cancer compared to non-Indigenous individuals. The incidence is four times greater in First Nations women compared to Caucasian women. A large proportion of patients with gallbladder disease live on reserves, land held by the Crown for the “use and benefit of First Nations” [8]. Individuals living on reserves and rural areas face disparities in accessing health services, due to their geographical and economical isolation.

One group that could benefit from prophylactic cholecystectomy is hemolytic disorder patients. Sickle cell disease patients have a higher incidence of pigment gallstones. Over half will develop biliary related complications in the first three to five years after diagnosis, due to repeated hemolytic crises [9]. Laparoscopic cholecystectomy should be considered in this subset of patients for two reasons. The first is that biliary complications of gallstone disease and vaso-occlusive crises have similar clinical presentations (abdominal pain, fever, leukocytosis, and deranged liver enzymes). Cholecystectomy reduces diagnostic uncertainty when a patient presents with the aforementioned symptoms. The second reason is that the early onset of gallstones in sickle cell patients increases the lifetime risk of biliary complications, so prophylactic cholecystectomy may be beneficial in this surgical subset [10].

Surgery for asymptomatic gallstones in patients undergoing transplantation may also be considered in the context of post-operative immunosuppression. Drugs such as Cyclosporine and Tacrolimus are lithogenic. When patients are immunosuppressed, the typical presentation of acute cholecystitis is blunted, leading to delays in diagnosis and an increase in morbidity and mortality. The aim of a prophylactic cholecystectomy is to remove potential septic foci that may cause complications in an immunosuppressed patient. The risk of wound complications from elective cholecystectomy must be weighed against the risks of developing cholecystitis and overt sepsis in the future.

Prophylactic cholecystectomy is not indicated in patients with diabetes mellitus or morbid obesity requiring bariatric surgery. The proportion of diabetics who develop biliary colic or gallstone related complications are equivalent to the general population, although they may present in a delayed fashion with emphysematous or gangrenous cholecystitis due to diabetic neuropathy [11]. Rapid weight loss after bariatric surgery (i.e. Roux-en-Y gastric bypass, sleeve gastrectomy or adjustable gastric band) can induce cholelithiasis, but the overall incidence of cholelithiasis post bariatric surgery is low at 9.7% [12]. This is similar to the 10–15% incidence in the general population [1].

## 4 Conclusion

This case report is the first in the English literature of an asymptomatic patient carrying a stone of these dimensions. While cholecystectomy is the standard of care for patients with symptomatic gallstones, there are specific subsets of asymptomatic patients who may be considered for surgery. Size and presence of stones increases the risk of gallbladder malignancy, fistulization, perforation, and cholecystitis. Patients who are transplant candidates or those with hemolytic disorders may be considered for prophylactic surgery. The risks and benefits of elective prophylactic cholecystectomy and future gallstone related complications should be discussed with the patient to enhance the shared decision making process in the management of their disease.

## Declaration of Competing Interest

No conflicts of interest.

## Sources of funding

No sources of funding for this case report.

## Ethical approval

No ethical approval necessary for this case report.

## Consent

Written informed consent was obtained for the publication of this case report and accompanying images.

## Author contribution

Dr. Chan was involved in the treatment of the patient, reviewed the patient medical records and literature and drafted the original manuscript. Dr. Pillay conceived the idea of the manuscript, was involved in the treatment of the patient. Dr. Sabaratnam performed pathological review of the patient’s surgical specimens. All authors reviewed, revised and approved the manuscript prior to journal submission.

## Registration of research studies

1. Name of the registry: Not applicable
2. Unique identifying number or registration ID: not applicable
3. Hyperlink to your specific registration (must be publicly accessible and will be checked): Not applicable.

## Guarantor

Dr Yagan Pillay is the guarantor.

## Provenance and peer review

Not commissioned, externally peer-reviewed.
